# Relationship between the effects of food on the pharmacokinetics of oral antineoplastic drugs and their physicochemical properties

**DOI:** 10.1186/s40780-019-0155-1

**Published:** 2019-12-03

**Authors:** Fukiko Omachi, Masaki Kaneko, Ryosuke Iijima, Machiko Watanabe, Fumio Itagaki

**Affiliations:** 1 Department of Clinical & Pharmaceutical Sciences, Faculty of Pharma Science, Teikyo University, 2-11-1, Kaga, Itabashi-ku, Tokyo, 173-8605 Japan; 2 Department of Medical & Pharmaceutical Sceinces, Faculty of Pharma Science, Teikyo University, 2-11-1, Kaga, Itabashi-ku, Tokyo, 173-8605 Japan

**Keywords:** Food effects, Oral antineoplastic drugs, Pharmacokinetics, Physicochemical properties, In silico

## Abstract

**Background:**

Food is known to affect drug absorption by delaying gastric emptying time, altering gastrointestinal pH, stimulating bile flow, increasing splanchnic blood flow, or physically interacting with drugs. Although food is known to affect the pharmacokinetics of oral antineoplastic drugs, the relationship between the effects of food and the physicochemical properties of drugs remains unclear.

**Methods:**

In this study, we surveyed the literature on three kinds of pharmacokinetic changes, AUC ratio, C_max_ ratio and T_max_ ratio, in the fasted and fed state for 72 oral antineoplastic drugs that were listed on the drug price standard in May 2018 in Japan. We further predicted the physicochemical properties from the 2D chemical structure of the antineoplastic drugs using in silico predictions.

**Results:**

As a result of analyzing the relationship between the effects of food and physicochemical properties, we found that compounds that show increased absorption in the fed state had higher logP and lower solubility in fasted-state simulated intestinal fluid (FaSSIF). However, compounds with delayed absorption had higher solubility in FaSSIF. Furthermore, as a result of decision tree analysis, it was classified as AUC increase with logP ≥4.34. We found that an AUC increase in the fed state did not occur with compounds with low lipid solubilities (logP < 1.59). From these results, it is predicted that 7 compounds out of the 24 compounds for which the effects of food are unknown are at risk for increased absorption in the fed state and that no increase in absorption would occur in 13 compounds.

**Conclusion:**

In this study, we found that drugs that will show increased absorption in the fed state and drugs for which absorption is not dependent on food can generally be predicted by logP. These results suggest that logP can be a useful parameter for predicting the effects of food on drug absorption.

## Background

Food is well known to affect drug absorption by delaying gastric emptying time, altering gastrointestinal pH, stimulating bile flow, increasing splanchnic blood flow, or physically interacting with drugs [1–3]. Furthermore, different foods, based on factors such as nutritional composition (high-protein, carbohydrate-rich, or high-fat meals), calorie content (low vs high calorie meals), volume, temperature and fluid ingestion, have distinct influences on the transit time, luminal dissolution, permeability and bioavailability of the drug product [4].

The Biopharmaceutics Classification System (BCS) is a scientific framework for classifying drug substances based on their aqueous solubility and intestinal permeability [5]. According to the BCS, drug substances are classified as four categories based on their solubility and intestinal permeability. Fisher et al. reported that drug-food interactions could generally be predicted based on the BCS class [6]. Class 1 drugs with high solubility/high permeability; high-fat meal will have no significant effect on drug bioavailability, Class 2 drugs with low solubility/high permeability; high-fat meal will increase drug bioavailability, Class 3 drugs with high solubility/low permeability; high-fat meal will decrease drug bioavailability, Class 4 drugs with low solubility-low permeability; it is difficult to predict what will occur [6, 7]. Gu CH et al. further improved the prediction of food effects by classifying drugs based on solubility, permeability and dose of a compound [8]. Although they analyzed 90 marketed compounds, only one oral antineoplastic drug was included in their models.

The number of oral antineoplastic drugs approved for manufacture in Japan has been substantially increasing [9]. In particular, remarkable increases in molecular target drugs, including many drugs affected by food, have occurred in recent years [10]. There are many drugs for which dietary conditions are defined in the usages described in the package inserts [11]. On the other hand, oral antineoplastic drugs that are not molecular target drugs include many drugs for which dietary conditions are not defined in the usage instructions. Since the therapeutic range and the toxic range are in close proximity for oral antineoplastic drugs, the effects of food must be considered when evaluating their varying pharmacokinetics. Although it is already known that food may affect the pharmacokinetics of oral antineoplastic drugs [12–14], the relationship between the effects of food and the physicochemical properties of the drugs remains unclear.

In this study, we review the pharmacokinetic changes caused by food in oral antineoplastic drugs and evaluate their relevance to the physicochemical properties of antineoplastic drugs by in silico predictions. In addition, we predicted the pharmacokinetic changes in drugs for which the effects of food are unknown using the physicochemical properties as indicators.

## Methods

### Investigation of oral antineoplastic drugs

We surveyed the literature on three kinds of pharmacokinetic changes, including the area under the curve of the drug concentration-time profile (AUC) ratio, the maximum serum concentration (C_max_) ratio, and the time at which C_max_ is observed (T_max_) ratio, in the fasted and fed state for 72 oral antineoplastic drugs that were listed on the drug price standard in May 2018 in Japan [15]. For drugs without ratio data in the literature, ratios were calculated from the medians or averages of the AUC, C_max_ and T_max_ values in the fasted or fed state. In addition, for drugs with data from several clinical trials, we selected data from high-fat meals when several data on meals were available and the closest data to those of the usage approved in Japan when data from several dosages and administration techniques were available. We analyzed the distributions of the AUC ratio, C_max_ ratio and T_max_ ratio and the relationships between the ln (AUC ratio) and ln (C_max_ ratio) using JMP® Pro 13.1.0 (SAS Institute Inc., Cary, NC, USA), which is a statistical analysis software, based on the collected information.

The magnitudes of the effects of food were classified based on the reported pharmacokinetic differences between the fed and fasted states. With regard to the AUC ratio, food effects were classified into 3 groups, the absorption increase group (AUC ratio > 1.25), the absorption invariant group (0.8 ≤ AUC ratio ≤ 1.25), and the absorption decrease group (AUC ratio < 0.8), in accordance with the variations in bioequivalence in the guidelines for bioequivalence studies of generic products (0.8–1.25) [16]. The T_max_ ratios were classified into 3 groups, the absorption time prolongation group (T_max_ ratio > 2.0), the absorption time invariant group (0.5 ≤ T_max_ ratio ≤ 2.0), and the absorption time shortening group (T_max_ ratio < 0.5).

### In silico prediction of the physicochemical properties of oral antineoplastic drugs

We predicted the following physicochemical properties from the 2D chemical structures of antineoplastic drugs by a prediction model using artificial neural network technology: octanol/water partition coefficient (logP); solubility in fasted-state simulated gastric fluid (FaSSGF), fasted-state simulated intestinal fluid (FaSSIF) and fed-state simulated intestinal fluid (FeSSIF) [17, 18]; and nonionized fraction at pH 6.8 (FUnion_6.8_) and pH 1.2 (FUnion_1.2_). These predictions were made using ADMET Predictor™ 8.1 (Simulation Plus, Inc., Lancaster, CA, USA), which is an ADMET physicochemical properties prediction software. For the accuracy of the logP predictions, the root mean square error (RMSE) was 0.314 log units, the mean absolute error (MAE) was 0.241 log units, and the *R*^*2*^ value was 0.971.

We analyzed the relationship between the known effects of food and physicochemical properties using JMP® Pro 13.1.0. We analyzed the bivariate relationship using AUC changes (AUC increase, invariance and decrease) as objective variables and logP and the solubility in FaSSGF, FaSSIF and FeSSIF as explanatory variables and compared the medians for all pairs using Steel-Dwass test. Similarly, we analyzed the bivariate relationship based on T_max_ changes (T_max_ prolongation, invariance and shortening) as objective variables and logP and the solubility in FaSSGF, FaSSIF, FeSSIF and FaSSIF/FeSSIF solubility ratio as explanatory variables and compared the averages using Welch’s test.

Based on the results of the analysis, a decision tree analysis was performed with the changes in AUC as the objective variables and logP as the explanatory variable. The criterion function by which nodes are split is the LogWorth statistic [LogWorth = (− 1)*ln (chi-squared *p*-value)], which is to be maximized. The division point of logP related to the increase in drug absorption by food was obtained. Furthermore, we predicted whether the absorption would increase for drugs for which the effects of food are unknown.

## Results

### Effects of food on the pharmacokinetics of oral antineoplastic drugs

**Fig. 1:**
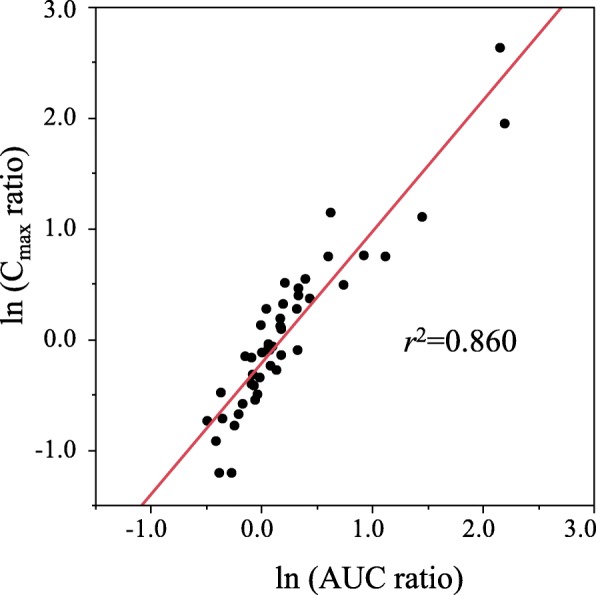
Bivariate relationship between ln (AUC ratio) and ln (C_max_ ratio)

Information on effects of food on the pharmacokinetics of 48 compounds (66.7%) out of the 72 investigated oral antineoplastic drugs was obtained. There were 30 compounds for which dietary conditions were defined in the usages or the precautions described in the package inserts; 15 compounds required postprandial administration, and the other 15 compounds required fasting administration (Table 1). The medians (maximum, minimum) of the AUC ratios, C_max_ ratios and T_max_ ratios were 1.08 (8.96, 0.61), 0.94 (13.97, 0.30) and 1.91 (3.92, 0.50), respectively. There was a positive correlation between ln (AUC ratio) and ln (C_max_ ratio) (*r*^2^ = 0.86) (Fig. 1).

**Table 1:** Usages in package inserts of oral antineoplastic drugs^a)^

Usage	Number of compounds
fasting	15
postprandial	15
before bedtime	1
certain conditions	2
unspecified	39
total	72

a) In addition to what is specified in the package inserts, conditions described in the precautions such as “avoid taking from 1 h before meal until 2 h after meal”, “avoid taking 1 h before and after meal” or “avoid taking from 1 h before meal until 2 h after meal when taking high fat meals” are also classified as “fasting”

Classification based on the type of the effect of food based on the AUC ratio resulted in 14 compounds in the absorption increase group, 26 compounds in the absorption invariant group, and 7 compounds in the absorption decrease group. Classification based on the T_max_ ratio resulted in 15 compounds in the absorption time prolongation group, 23 compounds in the absorption time invariant group, and no compounds in the absorption time shortening group. The compounds in the absorption increase group and absorption decrease group are shown in Table 2. The AUC increased by a factor of 8 or more due to food in the cases of bexarotene and abiraterone acetate. On the other hand, AUC decreased by approximately 60% due to food in the cases of capecitabine and afatinib.

**Table 2:** Oral antineoplastic drugs for which absorption is changed by food

Absorption	Generic name	Molecular weight	Usage	AUC ratio	C_max_ ratio	T_max_ ratio	logP	lnFaSSGF	lnFaSSIF	lnFeSSIF
absorption increase	bexarotene	348.48	postprandial	8.96	7.04	–	7.46	−8.28	−4.71	−1.02
	abiraterone acetate	391.55	fasting	8.59	13.97	1.00	5.40	−6.91	−4.71	−2.16
	lapatinib tosylate hydrate	943.49	fasting	4.25	3.03	2.53	5.18	− 4.71	−5.81	−4.83
	vemurafenib	489.93	fasting	3.05	2.12	1.88	5.17	−3.82	−4.02	−2.56
	pazopanib hydrochloride	473.98	fasting	2.51	2.14	1.51	3.46	−0.77	−5.30	−2.50
	erlotinib hydrochloride	429.90	fasting	2.09	1.64	1.04	3.13	−1.70	−5.81	−2.15
	ibrutinib	440.50	not mentioned	1.86	3.15	–	2.87	−3.38	−3.65	−2.98
	nilotinib hydrochloride hydrate	584.00	fasting	1.82	2.12	1.25	5.01	−4.02	−6.21	−2.49
	ceritinib	558.14	fasting	1.54	1.45	1.33	6.08	−4.96	− 4.96	−2.15
	regorafenib hydrate	500.84	postprandial	1.48	1.73	1.5	5.30	−5.30	−4.51	−2.45
	exemestane	296.41	postprandial	1.39	1.59	1.94	3.13	−2.85	− 2.75	−2.40
	bosutinib hydrate	548.47	postprandial	1.39	1.49	1.00	4.94	1.62	−4.61	−4.51
	vorinostat	264.32	postprandial	1.38	0.91	2.67	1.59	−0.93	− 0.028	−1.37
	gefitinib	446.91	postprandial	1.37	1.32	–	4.34	0.34	−4.42	−2.75
absorption decrease	everolimus	958.24	certain conditions	0.78	0.46	2.50	5.56	−6.91	−4.96	−0.71
	trametinib dimethyl sulfoxide	693.53	fasting	0.76	0.30	2.69	2.84	−5.52	−3.30	−2.33
	dabrafenib mesylate	615.67	fasting	0.70	0.49	3.00	4.71	−4.34	−4.96	−1.08
	sorafenib tosylate	637.03	fasting	0.69	0.62	1.00	5.07	−4.96	−4.27	−2.47
	ixazomib citrate	517.13	fasting	0.68	0.30	3.92	1.53	−5.12	0.30	−0.15
	capecitabine	359.35	postprandial	0.66	0.40	2.00	1.28	−0.068	−2.28	−0.042
	afatinib maleate	718.09	fasting	0.61	0.48	2.28	4.05	0.31	−3.41	−2.03

### In silico prediction of the physicochemical properties of oral antineoplastic drugs

**Fig. 2:**
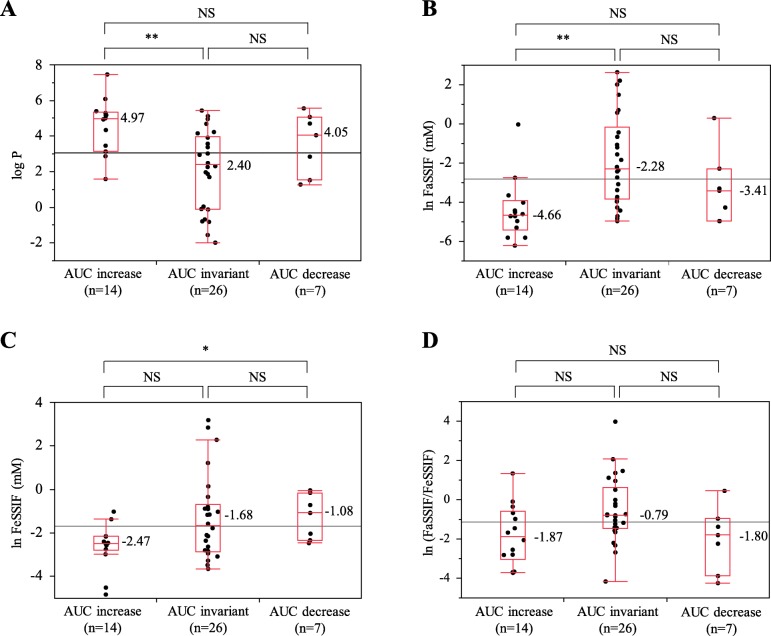
Relationship between the known food effects and physicochemical properties obtained by in silico predictions. **a** Relationship between AUC changes and logP. **b** Relationship between AUC changes and the solubility in FaSSIF. **c**Relationship between AUC changes and the solubility in FeSSIF. **d** Relationship between AUC changes and FaSSIF/FeSSIF solubility ratio. Steel-Dwass test. **:*P* < 0.01. *:*P* < 0.05. NS: not significant

**Fig. 3:**
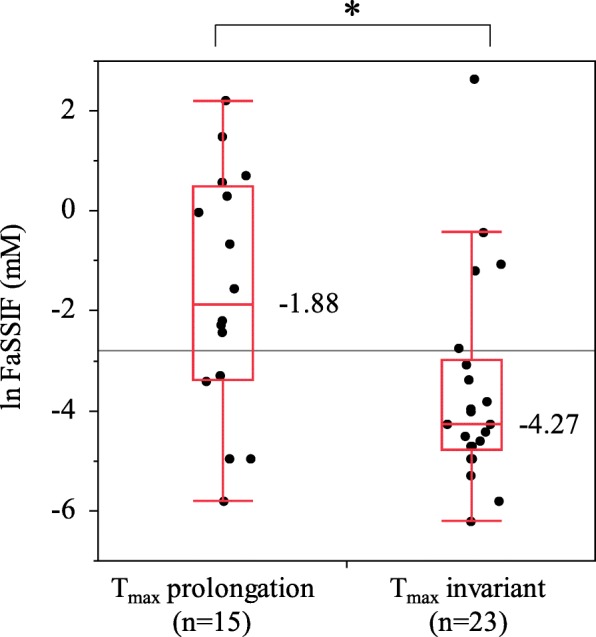
Relationship between T_max_ changes and the solubility in FaSSIF. Welch’s test. *:*P* < 0.05

Using JMP® Pro 13.1.0., we analyzed the relationship between the reported effects of food and the physicochemical properties obtained from in silico predictions. The bivariate relationship was analyzed using AUC changes as the objective variables and logP as the explanatory variable. The medians of the logP value (maximum, minimum) were 4.97 (7.46, 1.59) in the AUC increase group, 2.40 (5.44, − 1.99) in the AUC invariant group, and 4.05 (5.56, 1.28) in the AUC decrease group. The median in the AUC increase group was significantly higher than that of the AUC invariant group (*P* = 0.0054) (Fig. 2a). In the bivariate analysis of AUC changes and solubility in FaSSIF, the median of lnFaSSIF was − 4.66 in the AUC increase group, − 2.28 in the AUC invariant group and − 3.41 in the AUC decrease group. The median in the AUC increase group was significantly low than that of the AUC invariant group (*P* = 0.0013) (Fig. 2b). Similarly, in FeSSIF, the median of lnFeSSIF in the AUC increase group was lower than that of the AUC invariant group, although the difference was not significant (Fig. 2c). In the bivariate analysis of the changes in T_max_ and solubility in FaSSIF, the median of lnFaSSIF was − 1.88 in the T_max_ prolongation group and − 4.27 in the T_max_ invariant group (Fig. 3). The median in the T_max_ prolongation group was significantly higher than that of the T_max_ invariant group (*P* = 0.0129), and a similar trend was observed for FeSSIF. However, no significant difference was observed between the T_max_ prolongation group and the T_max_ invariant group in the bivariate analysis of the changes in T_max_ and logP. As described above, we found that compounds for which the absorption was increased by food had higher logP and lower solubilities in FaSSIF and FeSSIF and that compounds for which the absorption was decreased had higher solubilities in FaSSIF. On the other hand, no relationship between the effects of food and other physicochemical properties, such as the nonionized fraction, were observed.

Since a correlation was found between increased absorption by food and the values of logP, the decision tree analysis was performed with AUC changes as the objective variables and logP as the explanatory variable. The division point of logP related to the increase in drug absorption by food was obtained (Table 3). As a result, the division point of logP was 4.34, and it was classified as AUC invariant with logP < 4.34 and as AUC increase with logP ≥4.34 (the true rate was 77.5%). The false-positive rate and the false-negative rate were 15.4 and 35.7%, respectively. Furthermore, we found that an AUC increase due to food did not occur with compounds with lower lipophilicities (logP < 1.59). Based on these results, we were able to predict whether the absorption would increase for 24 compounds for which the effects of food are unknown (Table 4). We predicted that the risk of absorption increase due to food was high for 7 compounds with logP ≥4.34. All of these compounds had lower FaSSIF solubilities and were consistent with the characteristics of compounds for which the absorption was increased by food. On the other hand, we inferred that an absorption increase would not occur with 13 compounds with logP < 1.59. These compounds tended to show higher FaSSIF solubilities relative to compounds with logP ≥4.34.

**Table 3:** Decision tree analysis using AUC changes as objective variables and logP as the explanatory variable

	AUC increase	AUC invariant
logP ≥4.34	9	4
4.34 > logP ≥1.59	5	14
1.59 > logP	0	8

**Table 4:** Prediction of absorption changes in oral antineoplastic drugs for which the food effects are unknown

Prediction	Generic name	Usage	logP	lnFaSSGF	lnFaSSIF	lnFeSSIF
absorption increase (logP ≥4.34)	toremifene citrate	–	6.73	− 2.23	−4.34	−0.73
	tamoxifen citrate	–	6.64	−2.11	−4.20	−0.58
	tretinoin	postprandial	6.07	−6.91	−3.44	−1.13
	mitotane	–	6.06	−6.91	−4.42	−1.06
	mepitiostane	–	6.01	−7.83	−4.51	−2.56
	tamibarotene	postprandial	5.80	−5.81	−3.06	−1.13
	cytarabine ocfosphate hydrate	postprandial	4.56	−4.02	−5.81	−5.81
no prediction	medroxyprogesterone acetate	–	3.94	−5.30	−4.27	−2.59
	estramustine phosphate sodium hydrate	–	3.92	−3.30	−1.20	−1.56
	flutamide	postprandial	2.90	−2.69	− 2.15	−1.72
	anastrozole	–	2.67	−2.90	−3.54	−1.48
absorption invariant (logP < 1.59)	cyclophosphamide hydrate	–	0.77	2.47	1.96	2.84
	etoposide	–	0.76	−2.00	−1.80	−1.51
	sobuzoxane	–	0.46	−0.11	−3.04	−1.94
	tegafur	–	−0.12	1.35	1.49	2.28
	tegafur/gimeracil/oteracil potassium	postprandial	−0.12	1.35	1.49	2.28
	melphalan	–	−0.34	2.27	1.29	0.77
	mercaptopurine hydrate	–	−0.52	0.91	−1.14	−0.46
	fluorouracil	–	−0.81	1.49	1.66	2.67
	busulfan	–	−0.81	3.35	7.89	5.01
	doxifluridine	–	−1.08	2.50	3.57	3.36
	methotrexate	–	−1.11	3.02	1.06	−1.86
	hydroxycarbamide	–	−1.66	3.90	3.74	3.12
	ubenimex	–	−1.67	3.33	2.26	1.72

## Discussion

In this study, we analyzed variables correlated with the AUC ratio out of physicochemical properties obtained by in silico predictions, and it was suggested that drugs with high lipophilicities (logP values) and low intestinal solubilities (in FaSSIF and FeSSIF) had high risks of absorption increases due to food. This result is considered to be due to the solubility increase caused by the promotion of bile secretion by food [19]. Since the majority of tyrosine kinase inhibitors (TKIs) are substrates for drug transporters (e.g. ABCB1 and ABCG2) [7, 20], food may also inhibit drug transporters, thereby increasing drug absorption [10]. On the other hand, we predicted that an absorption increase would not occur for the compounds with high water solubilities. In the case of drugs with high intestinal solubility, there are risks of delaying the absorption rate.

In the decision tree analysis, the division point of logP related to increased drug absorption by food was calculated as 4.34. In support of this finding, a previous study that predicted the effects of fed-state intestinal contents on drug dissolution showed that hydrophobic drugs with logP > 4 showed a significant increase in solubility in FeSSIF [18]. It was also reported that increase of solubilization by bile acids would not occur in drugs with a logP< 2 [3, 21]. When the division point of logP was 4.34, 64% (9 out of 14) of true positives (AUC increase) could be accurately predicted, while 36% were predicted as false negatives. In other words, logP≥4.34 provides a high likehood of being an AUC increase drugs, whereas 36% of AUC increase drugs have the properties with logP < 4.34. Total 85% (22 out of 26) of true negatives (AUC invariant) could be accurately predicted, while only 15% were predicted as false positives. That means that AUC invariant drugs are almost in logP < 4.34.

Based on the literature results, we found that the usages described in the package inserts in most drugs for which the absorption increased or decreased due to food were defined as postprandial administration, fasting administration, or other specific conditions. On the other hand, for the drugs for which no clinical trial data on the effects of food are available, 7 compounds were predicted to have risks of absorption increases by decision tree analysis, and meal conditions were defined in the usages in the package inserts for only 3 of these compounds. Since the absorption may be increased by food in drugs with logP ≥4.34, food effects should be considered even in the cases of drugs that have no clinical trial data on food effects. In this study, we focused on the pharmacokinetic changes caused by food in oral antineoplastic drugs and evaluate their relevance to logP values. A logP value, which indicates lipophilicity, is a frequently used parameter in correlation with membrane permeability [22–25] and is a popular index for Japanese pharmacists. The logP value of each antineoplastic drug is easily available on the drug package insert, and is easy to evaluate for pharmacist. The BCS classification has been used to evaluate food-drug interactions in the development stage of pharmaceuticals [26, 27], however, the index is not popular among clinical pharmacists in Japan to date. Additionally, the identification of “highly soluble” and “highly permeable” for BCS are not simple [26, 27]. Therefore, we believe that the simple prediction of drug-food interaction by the logP values obtained from the results of this study is useful for clinical pharmacists.

In this study, we found significant differences between compounds with food-induced absorption increases and food-invariant absorption, and between those with food-induced absorption time prolongation and food-invariant absorption times. In addition, we found some common trends in these compounds based on their structures. There was no significant difference between the compounds for which the absorption is decreased by food and those for which the absorption is increased or invariant. Although their molecular weights tend to be large, there were only 7 compounds for which absorption decreased due to food, making it difficult to conduct further evaluations. Additionally, further evaluation of the relationship between food and the physicochemical properties is required because drug administration tests are not performed under the same conditions and the contents of the meal ingested can vary. It is difficult to verify our predictions in clinical trials, we are trying to build a more accurate *in slico* classification model, using an artificial neural network (ANN) as an alternative method to verify our predictions.

## Conclusion

In this study, we found that the antineoplastic drugs for which absorption increases or does not change due to food can be generally predicted by their logP values. This suggests that we should implement pharmaceutical management with regard to meals and the timing of administration using logP as an index and considering the characteristics of drugs such as the narrowness of their therapeutic and toxic ranges.

## Data Availability

All data generated or analyzed during this study are included in this published article.

## Abbreviations

2D: Two-dimensional
AUC: Area under the curve of the drug concentration-time profile
C_max_: Maximum serum concentration
FaSSGF: Fasted-state simulated gastric fluid
FaSSIF: Fasted-state simulated intestinal fluid
FeSSIF: Fed-state simulated intestinal fluid
logP: Partition coefficient
MAE: Mean absolute error
RMSE: Root mean square error
T_max_: The time at which C_max_ is observed
